# Nano-Hydroxyapatite toothpaste of rice field snail shell combined with basil leaf extract as a remineralizing and antibacterial agent to prevent dental caries

**DOI:** 10.4317/jced.62073

**Published:** 2024-11-01

**Authors:** Wienda Cinta Aliyyu, Farahdika Ardelia Riva, Sabrina Majesta Putri Anabel, Irfan Dwiandhono, Rinawati Satrio, Dwi Nur Indah Sari

**Affiliations:** 1 Faculty of Medicine, Universitas Jenderal Soedirman. Banyumas, Central Java, Indonesia; 2 Dental and Oral Hospital of Universitas Jenderal Soedirman; 3 Center of Applied Science for Pharmaceutical and Health, Universitas Jenderal Soedirman

## Abstract

**Background:**

This study aimed to compare rice snail shell nano-hydroxyapatite (nano-HAp) toothpaste combined with basil leaf extract and 1450 ppm fluoridated herbal toothpaste as remineralizing and antibacterial agents.

**Material and Methods:**

Experimental toothpastes were prepared with different concentrations of nano-HAp (5% nano-HAp, 10% nano-HAp) and a fixed concentration of basil leaf extract (5% extract). 24 bovine tooth enamel samples were divided into 4 treatment groups. After 6 days of pH cycling, surface microhardness was tested using Vickers Microhardness Test and microporosity was observed using Scanning Electron Microscopy (SEM). The pitting diffusion method was used to test the antibacterial activity of toothpaste against Streptococcus mutans and Lactobacillus acidophilus.

**Results:**

Enamel surface microhardness showed an increase with increasing nano-HAp concentration, while fluoride toothpaste showed the highest level of surface microhardness. Microporosity observation showed the disappearance of microporosity as nano-HAp concentration increased, while fluoride toothpaste still left microporosity. Antibacterial activity of the toothpaste showed that the mean inhibition zone of each group was not significantly different with a very strong inhibition zone.

**Conclusions:**

Rice snail shell nano-HAp toothpaste combined with 5% basil leaf extract is effective as a remineralizing agent by reducing the level of tooth enamel microporosity but cannot match 1450 ppm fluoride toothpaste in increasing surface hardness. In addition, nano-hydroxyapatite toothpaste of paddy field snail shell combined with 5% basil leaf extract is effective as an antibacterial agent for preventing dental caries because it has antibacterial activity equivalent to 1450 ppm fluoride herbal toothpaste.

** Key words:**Nano-hydroxyapatite, basil leaf extract, toothpaste, remineralization, antibacterial.

## Introduction

Dental caries is a global dental health problem recorded in The Global Burden of Disease in 2019, with a 43% prevalence in deciduous teeth, predominantly in South-East Asia (135 million cases). For permanent teeth, the prevalence is 29%, with the highest case numbers in South-East Asia (526 million) and the Western Pacific (464 million) (1). Dental caries is characterized by demineralization of tooth enamel, followed by pulp tissue damage and spread of infection to periapical tissues. Dental caries can cause pain and disturb in chewing, speech and aesthetic functions (2). The main cause of dental caries is cariogenic bacteria that produce acid from the fermentation process of food debris or drink in the mouth that create acidic conditions and cause the dissolution of tooth minerals. Dental caries are caused by cariogenic bacteria on the tooth surface that ferment free sugars in food or drinks into acid. Sustained acidic conditions can induce tooth demineralization and cause enamel porosity, initiating caries development (3).

Caries can be prevent with brushing the teeth using toothpaste that contains fluoride as a remineralizing agent (4). However, the effectiveness of *fluoride* depends on availability of calcium and phosphate ions in saliva to form acid-resistant fluorapatite. The remineralization effect of *fluoride* can be enhanced by increasing its dose, but there is a limit to the fluoride dose that achieves the maximum remineralization level (5). This dose limitation is related to the side effects of *fluoride*, which can lead to dental fluorosis and toxicity (6). Therefore, the new remineralizing agents that are comparable or more effective than *fluoride* is needed. Hydroxyapatite (HAp), a biocompatible calcium phosphate mineral, in nano size can promote remineralization in early carious lesions by filling the enamel porosity caused by caries process, without toxicity risk (7). Nano-hydroxyapatite (nano-HAp) promotes remineralization by providing calcium phosphate ions in demineralized areas and binding strongly to the enamel surface in the form of an apatite layer (8).

Nano-hydroxyapatite (nano-HAp) can be produced from natural materials such a rice field snail shells which are rich in calcium carbonate (95-99%). Rice field snail (*Pila ampullacea*) is an agricultural pest that is quite abundant and has not been optimally utilized. Calcium carbonate compounds in the shells of rice field snail can be calcined into calcium oxide (CaO) which is the main ingredient for hydroxyapatite synthesis (9).

Antibacterial content in toothpaste is also important to prevent dental caries by killing cariogenic bacteria. Basil leaf (*Ocimum sanctum L*.) is a natural ingredient with antibacterial potential due to its secondary metabolite compounds. Basil leaf extract in concentrations of 5% and 10% is effective against cariogenic bacteria, such as *Streptococcus mutans* and *Lactobacillus acidophilus* bacteria (10).

Based on the above description, the development of nano-HAp toothpaste of rice field snail shells combined with basil leaf extract is needed for preventing dental caries. This study aims to determine the potential of nano-HAp toothpaste of rice field snail shell combined with basil leaf extract as a tooth remineralization agent and its antibacterial activity against *Streptococcus mutans* and *Lactobacillus acidophilus*.

## Material and Methods

1. Preparation of basil leaf extract and phytochemical screening

Basil (*Ocimum sanctum L*.) leaves were sorted, cleaned, dried, and ground to obtain simplicia powder. Then, extraction was carried out by maceration method in 4 L of 70% ethanol solvent for 3x24 hours. The results of the macerate were filtered and evaporated to obtain a thick extract (11). Then, the qualitative phytochemical screening was conducted to assess the presence of flavonoids, saponins, tannins, triterpenoids, and alkaloids (12).

2. Synthesis and characterization of rice field snail shells nano-hydroxyapatite (nano-HAp) 

Rice field snail shells were cleaned, dried, and calcined at 1000°C for 5 hours at a heating rate of 5°C/minute into calcium oxide (CaO). Then, CaO was converted to calcium hydroxide ( Ca(OH) 2) by suspending it in 100 ml of distilled water and stirring with a magnetic stirrer (300 rpm; ± 25 °C). Phosphoric acid solution (H3PO4) was added gradually at 5 ml/min for 1 hour until a semi-liquid solution was formed. The semi-liquid solution was then irradiated in a 400 watt microwave for 45 minutes to produce nano-HAp. The nano-HAp was ground and filtered with a mesh to obtain nano-HAp powder. Then, the synthesized nano-HAp were characterized using PSA (*Particle Size Analyzer, Beckman Coulter’s LS 13 320 XR*) to determine particle size and XRD (*X-Ray Diffraction*) analysis using *Panalytical AERIS* (CuKα, 40kV, 15mA) to identify the crystal structure of hydroxyapatite (13).

3. Preparation of toothpaste

Toothpaste was prepared in five steps with the formulas listed in  Table 1. In step 1, nano-HAp powder was dispersed in aquabides with a 200 rpm *magnetic stirrer* for 50 minutes. Then, sodium benzoate was added with stirring until the mixture was homogeneous. Step 2, xanthan gum and a small portion of glycerin were homogenized in a separate cup. Step 3, the mixture of step 2 was mixed into the solution of step 1, then it stirred until homogeneous. Step 4, the remaining glycerin was mixed with sorbitol in a separate container. Then, the mixture from step 4 was added into the mixture from step 3 and stirred until homogeneous. Step 5, calcium carbonate (CaCO3) was added little by little while stirring until homogeneous. At this step, basil leaf extract is added. In the last step, sodium lauryl sulfate (SLS) was added to the mixture and stirred until homogeneous (14).

4. Tooth sample preparation

Bovine incisive tooth crowns free of decay lesions were cleaned. It cut using a *diamond disc* into enamel blocks (6mm x 6mm x 2mm), and stored in 0.1% thymol solution. Then, the tooth blocks were embedded in self cure acrylic resin with the buccal surface exposed and parallel to the horizontal plane. The buccal surface was polished with sequential abrasive papers (600, 800, 1000, 1500 grit). A 4 mm x 4 mm enamel window was made in the center of the sample surface, while other areas were coated with nail varnish for acid resistance (15).

5. Dynamic *pH cycling model* and toothpaste application

Test and control toothpaste (commercial herbal toothpaste with *fluoride* content of 1450 ppm) were diluted with artificial saliva solution (1:3) using a magnetic stirrer. The *pH cycling model* treatment was carried out by mimicking the exposure of the demineralization-remineralization cycle in the oral cavity to tooth samples. Tooth samples were demineralized for 16 hours at 37°C to induce artificial caries lesions, then toothpaste was applied. The pH cycling exposure was carried out by immersing samples in artificial saliva for 20 hours, demineralization solution for 2 hours, and toothpaste application twice a day for 2 minutes with a soft-bristled toothbrush for 6 days (15).

6. Evaluation of remineralization activity by *Vickers Microhardness Test and Scanning Electron Microscopy* (SEM) Evaluation

Tooth samples of each group (n=5) were tested for surface microhardness using a *Micro-vickers Hardness Tester* machine with a load of 100 grams for 30 seconds at 1 surface point leaving a rhombic indentation trace. The hardness value was generated by calculating the diagonal distance of the indentation marks. The tooth samples from each group (n=1) were examined for microporosity using Scanning Electron Microscopy at 7500× magnification after being coated with gold (16).

7. Evaluation of antibacterial activity of toothpaste

Evaluation of antibacterial activity of toothpaste was carried out using the well diffusion method against *Streptococcus mutans* ATCC 25175 and *Lactobacillus acidophilus* ATCC 4358 bacteria. The evaluation begins with the culture of *S.mutans* bacteria using De Man Rogosa Sharp Agar (MRSA) media and *Lactobacillus acidophilus* using Brain Heart Infusion Agar (BHIA) media. Then, the suspensions of *Streptococcus mutans* and *Lactobacillus acidophilus* were inoculated on the surface of Natrium Agar (NA) media evenly on different plates. A total of 1.2 grams of toothpaste preparation was put into different wells and incubated for 24 hours at 37°C. After 24 hours, the clear zone around the wells was measured for the presence of antibacterial activity (10).

## Results

1. Extraction results and phytochemical screening of extracts

The yield of basil leaves extraction was 19.02%. Phytochemical screening results showed that basil leaf extract contains of flavonoids, saponins, tannins, triterpenoids, and alkaloids with details in  Table 2.

2. Characterization of nano-HAp samples from rice field snail shells

**Figure 1:**
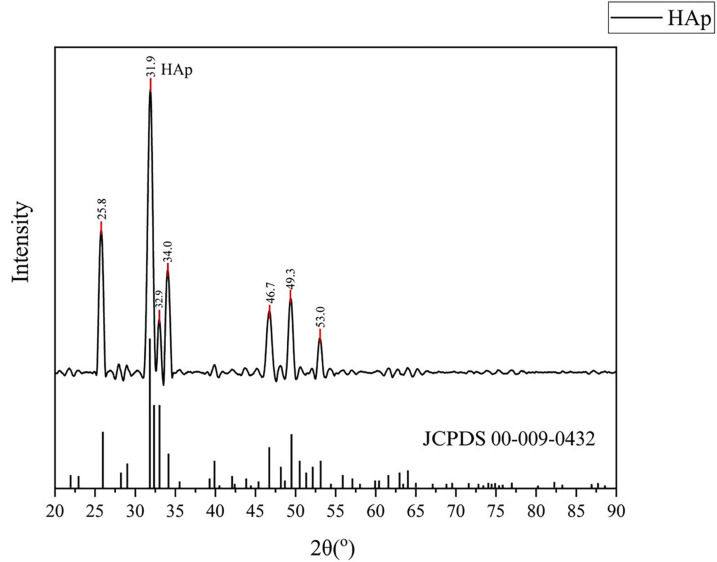
XRD pattern of nano-hydroxyapatite sample (top). Reference standard hydroxyapatite JCPDS No. 00-009-0432.

**Figure 2:**
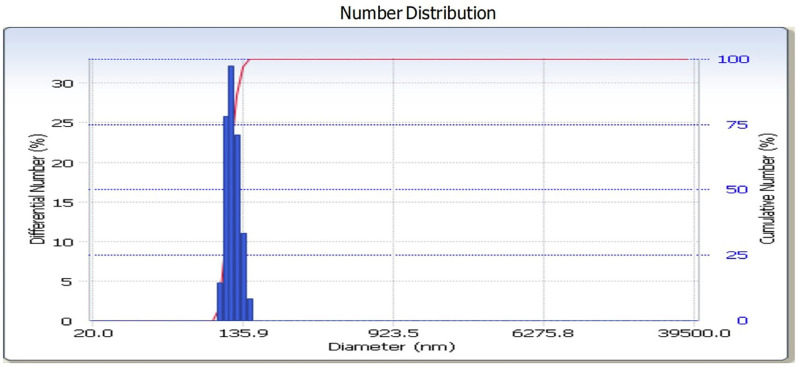
Particle size analysis (PSA) test results of nano-HAp samples.

Rice field snail shells nano-HAp samples were tested with XRD (X-ray Diffracton) to analyze crystal stucture of nano-HAp and the results are presented in the diffractogram (Fig. 1). The diffractogram showed that the highest peak of hydroxyapatite in this study at 31.91°. Particle size testing of nano-HAp samples also conducted in this study using Particle Size Analysis (PSA). The results showed that the particles had a size range of 100-135.9 nm, with an average of 118.7 nm (Fig. 2).

3. Evaluation results of remineralization activity 

- Vickers Microhardness Test Results

The microhardness test results indicated that enamel surface microhardness increased with higher nano-HAp concentrations but the highest results was in the *fluoride* toothpaste group (*p*<0.001) ( Table 3).

- Scanning Electron Microscopy (SEM) Results

**Figure 3:**
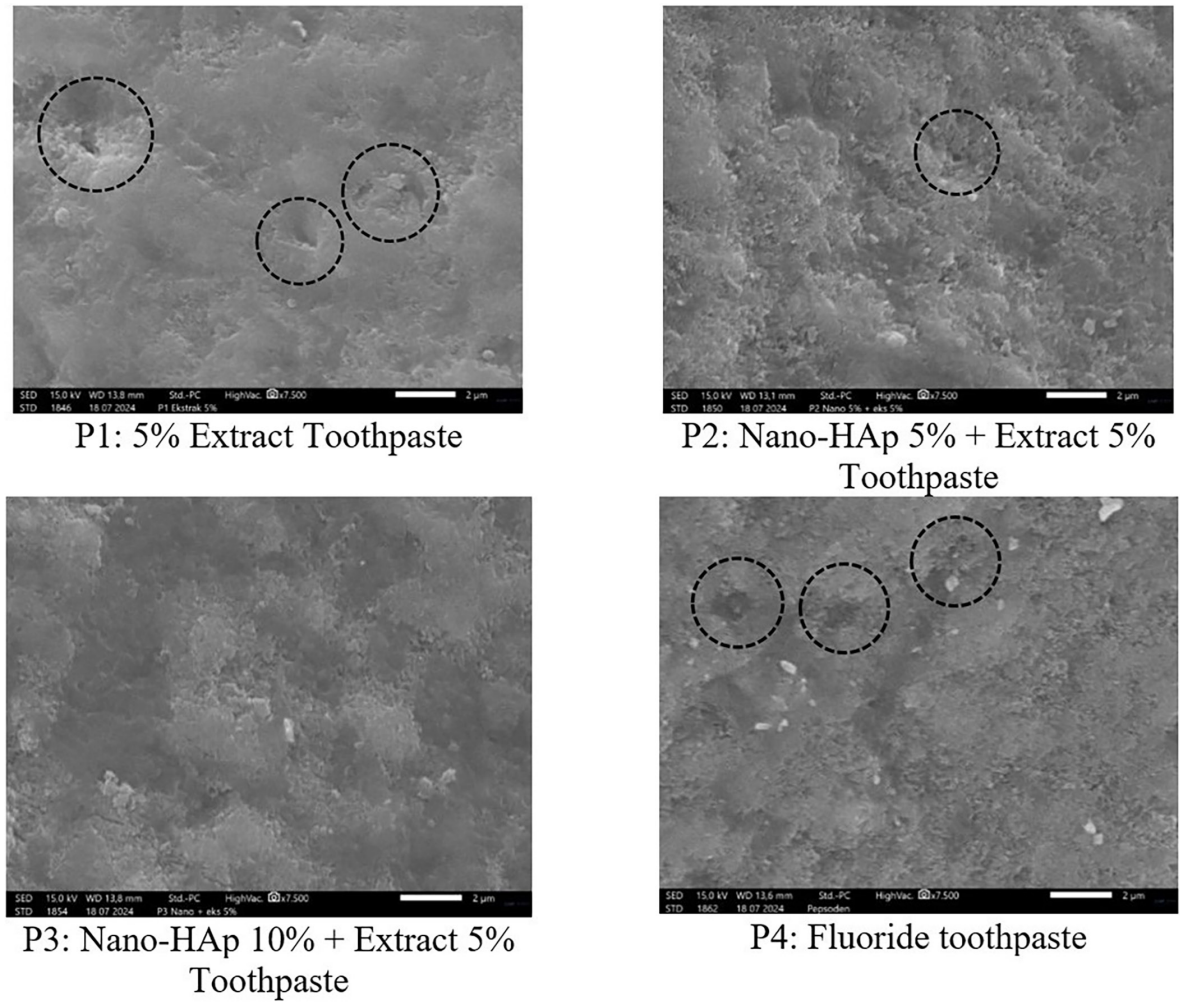
Observation of enamel microporosity using SEM (magnification 7500). Marked area referred to microporosities.

The SEM microporosity test at 7500x magnification revealed that microporosity persisted in the 5% basil leaf extract group (without nano-HAp). However, in the 5% and 10% nano-HAp toothpaste treatment groups, surface microporosity decreased as the nano-HAp concentration increased, resulting in a smoother surface at the 10% concentration (Fig. 3).

4. Antibacterial evaluation results of toothpaste 

The results of antibacterial activity showed that average inhibition zone in each group was not significantly different (*p*>0.05) and indicated a very strong antibacterial activity according to Davis & Stout category ( Table 4).

## Discussion

This study was used basil leaves as an addition to the antibacterial content of toothpaste. The plant sample used was basil leaves from the species *Ocimum sanctum L*., *synonym Ocimum tenuiflorum L*. Extraction of basil leaves was carried out by maceration method. The maceration method is an extraction method without heating so that damage to the active plant compound content can be minimized (11). The extract of basil leaves underwent qualitative phytochemical screening to identify the presence of bioactive compounds. This screening method is relatively easy and fast to do by detecting changes in the color of the extract (12). The results of qualitative phytochemical screening showed that basil leaf extract contains flavonoids, saponins, tannins, triterpenoids, and alkaloids with details in  Table 2.

The nano-HAp synthesis of rice field snail shells in this study was carried out by *microwave assisted wet precipitation* method. This method has the advantages of short reaction time, *rapid heating*, and maximum purity of nano-HAp results (17). The synthesis of nano-HAp from eggshell with the same method has been done before by Sari and colleagues and the best result of nano-HAp is at 400 watts microwave irradiation for 45 minutes (13). Our study used *microwave* irradiation with the same power and duration as the previous study (400 watts, 45 minutes) to produce 28 grams of nano-HAp.

The crystalline components of the nano-HAp sample of rice field snail shells were identified and analyzed for structure by XRD. The diffractograms presented in Figure 1 showed that the most intense Bragg reflection of nano-HAp occurs in the (211) plane. These pattern closely match the reference model for pure hydroxyapatite (JCPDS No. 00-009-0432) and there is no detecTable impurities in samples accordance to peak in XRD pattern. This result suggested that our sample consist of pure of hydroxyapatite. Moreover, our study demonstrated that the synthesized nano-HAp exhibits high crystallinity, as evidence by a sharp peak observed at diffraction angle (2θ) of 31.9°. The sharp peaks observed in this study are consistent with previous research using the same synthesis method, which also produced nano-HAp with a sharp peak at 2θ = 31.793° (13). Previous studies by Juntavee and colleagues reported that crystallinity of nano-HAp could influence mechanical properties. Higher crystallinity can give better mechanical properties, which can contribute to improved enamel strength after remineralization (18).

In addition to the crystallinity of the hydroxyapatite (HAp) nanoparticles, Nano-HAp size is also believed to impact the remineralization activity. The hydroxyapatite particle size in this study ranged from 100 to 135.9 nm, with an average of 118.7 nm. Nanoparticle size is generally less than 1 micron or 1000 nm, but nanoparticles below 500 nm have better characteristics (19). Nano-HAp with less than 200 nm in size is suitable for repairing nano-sized defects due to acidic erosion at the enamel surface (20).

The remineralization activity in this study was tested by enamel surface microhardness and microporosity. Enamel surface hardness test was conducted using Vickers Microhardness Tester. The results showed that the lowest mean enamel surface hardness was found in the 5% basil extract toothpaste group without nano-HAp (35.0 VHN), followed by the nano-HAp group (5% and 10%) and the highest score in the fluoride toothpaste group (Fig. 3).This result showed that the hardness of enamel surface increased as the concentration of nano-HAp increased. Nano-HAp remineralizes lesions by forming a homogeneous apatite layer on the enamel surface through chemical bonding with natural apatite crystals which can further affect enamel hardness (21). Previous studies have also investigated the combination of nano-HAp and natural ingredients. The findings indicate that there was a synergistic effect between nano HAp and natural components (*Galla chinensis*) in the remineralization process, as evidenced by increased mineral deposition within the lesion body, a significant reduction in lesion depth, and an improvement in microhardness (22).

The highest enamel surface microhardness in this study was found in the *fluoride* toothpaste group. This result was also found in a previous study by Aidaros and colleagues which showed that the *fluoride*-containing toothpaste treatment group showed the highest surface microhardness. This can be attributed to the formation of a fluorapatite layer on the enamel surface. This fact is also supported by the results of previous studies which show that enamel applied with toothpaste with a combination of nano-hydroxyapatite and *fluoride* has a higher surface microhardness than toothpaste with nano-hydroxyapatite without *fluoride* (23). Other study by Juntavee and colleagues reported different findings. They reported that administration of nano-Hap toothpaste, provides a remineralization effect by increasing surface hardness and showed better than *fluoride* group (18). The discrepancy between our findings and Juntavee and colleagues’s research can likely be attributed to the variation in concentrations of nano-HAp used. While Juntavee and colleagues utilized concentrations of 20% and 30%, our study examined lower concentrations of only 5% and 10%. Moreover, remineralization effectiveness relies on the specific geometry, enamel pore size, degradation rate of nano-HAp, and size of nano-HAp, with smaller particle sizes being preferable (24). nano-HAp particles sized between 20–50 nm are more effective at penetrating beneath the surface of enamel due to their nano-scale dimensions (24). This might also causes microhardness result in the nano-HAp group in this study were still not able to match those of the *fluoride* group. Different from other studies which reported that 10% nanohydroxyapatite was the optimal concentration for effective remineralization (24,25)

Evaluation of enamel surface microporosity in this study was evaluated using Scanning Electron Microscopy (SEM). Observations at 7500x magnification showed that the sample group treated with 5% basil leaf extract toothpaste without nano-HAp still had microporosity. Meanwhile, observations of the sample group treated with nano-HAp toothpaste (5% and 10%) showed a surface that did not have microporosity gaps as the nano-HAp concentration increased. This indicates the ability of remineralization by nano-hydroxyapatite to fill small microporosities and depressions on the enamel surface, with the effectiveness of remineralization increasing with increasing concentration (25). Small hydroxyapatite nanoparticles are able to penetrate the porosity of enamel rods that are 4-8 μm in diameter and repair the damaged tooth microstructure (26). The *fluoride* toothpaste group showed a thin layer of fluorapatite that still left microporosity cavities indicating incomplete *fluoride* remineralization ability on the entire surface.

Upon the initiation of demineralization, the enamel prism junctions are enlarged, and the interprismatic structure destruction is observed, allowing nano-hydroxyapatite (nano-HAp) penetration into the interprismatic enamel space. Additionally, certain matrix proteins embedded in the enamel’s organic component act as scaffolds, allowing nano-HAp to be easily deposited within the nano-gaps, capturing minerals to form apatite layer (7).

Nano-hydroxyapatite directly provides calcium and phosphate ions needed for enamel remineralization, which integrate into the enamel to repair damage from demineralization. Unlike *fluoride*, which depends on the presence of these ions in saliva, nano-hydroxyapatite is effective even in patients with low saliva production, making it a suitable option for those with salivary issues. Additionally, nano-hydroxyapatite is considered safer for long-term use as it carries no risk of fluorosis, making it particularly beneficial for vulnerable populations, such as children during tooth development (27).

The criteria for antibacterial activity of toothpaste in this study were expressed by the diameter of the inhibition zone against *Streptococcus mutans* and *Lactobacillus acidophilus*. According to Davis & Stout (1971), an inhibition zone of less than 5 mm is considered weak, 5-10 mm is considered moderate, 10-20 mm is considered strong, and more than 20 mm is considered very strong (28). Based on these criteria, the results showed that toothpaste containing 5% basil extract in each type of toothpaste (P1, P2, P3) has antibacterial activity with a very strong inhibition zone that is comparable to commercial herbal *fluoride* toothpaste. Meanwhile, the antibacterial activity of P1, P2, P3 is not significantly different because they contain similar concentrations of basil leaf extract. Previous study showed that *Ocimum sanctum* leaf extract exhibits antibacterial activity against *Streptococcus mutans* and *Lactobacillus acidophilus*. The very strong antibacterial activity of toothpaste is attributed to the content of basil leaf extract which has antibacterial active compounds, such as flavonoids, saponins, tannins, triterpenoids, and alkaloids (10).

Previous study conducted by Florea and colleagues demonstrated that combining nano-hydroxyapatite (nano-HAp) with birch extract toothpaste produces a synergistic effect, enhancing both antibacterial and remineralization properties. This synergy is likely due to the active compounds present in the birch extract. The research found that nano-HAp alone toothpaste was less effective than either the extract-only toothpaste or the combination of nano-HAp and extract toothpaste. However, the relative effectiveness of the extract-only treatment versus the combination treatment varied depending on the specific bacterial species being targeted (14).

*Streptococcus mutans* and *Lactobacillus acidophilus* play significant roles in the development of dental caries. *S. mutans* is known for its ability to form biofilms and produce acid, leading to caries formation. Meanwhile, *L. acidophilus*, though not as adept at forming biofilms, thrives in the acidic conditions created by *S. mutans* and further supports caries progression by continuing to produce acids. Both bacteria are essential targets for antibacterial agents in the toothpaste, which aim to inhibit their growth and acid production, thereby helping to prevent dental caries (29).

These findings provide valuable insights into enamel remineralization using nano-hydroxyapatite, but some limitations should be acknowledged. The main limitation of this study is the lack of biological processes in the oral cavity, especially within the dental plaque environment, which may hinder the remineralization process of the product being studied. Additionally, we recommend investigating the antibacterial effects of toothpaste formulations without sodium benzoate as a preservative. This approach will allow for a more accurate assessment of the inherent antibacterial properties of the plant extracts, as sodium benzoate might contribute to antibacterial activity that could mask or alter the extract’s natural effects. Long-term clinical trials and formulations with different nano-HAp concentrations are also recommended to enhance effectiveness. Furthermore, the study was conducted in controlled laboratory settings that may not fully mimic the complex oral environment, and it primarily focuses on short-term effects, observing enamel changes over a limited period, thus, further research into long-term impacts is also advised.

## Conclusions

The conclusions of this research are.

1. Nano-hydroxyapatite toothpaste made from rice field snail shells, combined with 5% basil leaf extract, effectively increases enamel hardness as a remineralizing agent, although it is not as effective as 1450 ppm fluoride toothpaste.

2. Nano-hydroxyapatite toothpaste made from rice field snail shells, combined with 5% basil leaf extract, effectively reduces enamel microporosity better than 1450 ppm fluoride toothpaste.

3. Nano-hydroxyapatite toothpaste made from rice field snail shells combined with 5% basil leaf extract is effective as an antibacterial agent, showing antibacterial activity comparable to commercial herbal fluoride toothpaste.

## Figures and Tables

**Table 1:** Nano-HAp toothpaste formula with basil leaf extract combination.

No.	Formula Composition	Amount per 100 grams (%)			Function
		P1	P2	P3	
1	Calcium carbonate	29	29	29	Abrasive material
2	Glycerin	18	18	18	Humectants
3	Sorbitol	12	12	12	Sweetener
4	Xanthan gum	2	2	2	Thickener
5	SLS	1	1	1	Surfactants
6	Na Benzoate	0,2	0,2	0,2	Preservatives
7	Aquabides	29,8	24,8	19,8	Solvent
8	Ethanol	3	3	3	Solvent
9	Basil Extract	5	5	5	Active ingredients
10	Nano-HAp	-	5	10	Active ingredients
	Total	100	100	100	

P1 (5% basil leaf extract toothpaste), P2 (5% paddy field snail shell nano-HAp toothpaste + 5% basil leaf extract), P3 (10% paddy field snail shell nano-HAp toothpaste + 10% basil leaf extract), P4 (Positive control; 1450 ppm herbal fluoride toothpaste).

**Table 2:** Phytochemical screening results of basil leaf extracts.

No.	Phytochemical	Test	Observation	Result
1	Flavonoids	Shinoda test	yellow color on the amyl alcohol layer	+
2	Saponins	Foam test	stable foam	+
3	Tannins	Ferric chloride test	blackish green color	+
4	Triterpenoids	Liebermann-Burchard test	red color	+
5	Alkaloids	Mayer's test	white sediment	+

**Table 3:** Average results of surface microhardness.

No.	Treatment	n	Mean ± SD	P-value
1.	P1 (5% Basil Extract)	6	35,20 ± 1,42	0,000*
2.	P2: (Nano-HAp 5%+Basil extract 5%)	6	38,80 ± 1,35	
3.	P3 (Nano-HAp 10%+Basil extract 5%)	6	47,40 ± 1,91	
4.	P4 (Commercial Fluoride Toothpaste)	6	65,20 ± 2,03	

n: number of samples, SD: standard deviation, * significantly different with one-way anova test

**Table 4:** Antibacterial test results of toothpaste.

Bacteria	Mean ± SD					Zone of Inhibition Category	p value*
	n	P1	P2	P3	P4		
Streptococcus mutans	5	25,00 ± 0,37	24,82 ± 0,60	23,88 ± 0,32	24,32 ± 0,51	Very strong	0,350
Lactobacillus acidophillus	5	25,66 ± 1,02	24,78 ± 1,04	24,84 ± 0,83	25,40 ± 0,94	Very strong	0,896

n: number of samples, SD: standard deviation, * p value with one-way anova test. P1 (5% basil leaf extract toothpaste), P2 (5% rice field snail shells nano-HAp toothpaste + 5% basil leaf extract), P3 (10% rice field snail shells nano-HAp toothpaste + 10% basil leaf extract), P4 (Positive control; 1450 ppm commercial herbal fluoride toothpaste).

## Data Availability

The datasets used and/or analyzed during the current study are available from the corresponding author.
